# Vertigo and dizziness in adolescents: Risk factors and their population attributable risk

**DOI:** 10.1371/journal.pone.0187819

**Published:** 2017-11-13

**Authors:** Filipp M. Filippopulos, Lucia Albers, Andreas Straube, Lucia Gerstl, Bernhard Blum, Thyra Langhagen, Klaus Jahn, Florian Heinen, Rüdiger von Kries, Mirjam N. Landgraf

**Affiliations:** 1 Department of Neurology, University Hospital, LMU, Munich, Germany; 2 German Center for Vertigo and Balance Disorders, LMU, Munich, Germany; 3 Institute of Social Paediatrics and Adolescents Medicine, Division of Epidemiology, LMU, Munich, Germany; 4 Department of Paediatric Neurology and Developmental Medicine, Hauner Children’s Hospital, LMU, Munich, German*y*; 5 Department of Neurology, Schön Klinik Bad Aibling, Bad Aibling, Germany; Taipei Veterans General Hospital, TAIWAN

## Abstract

**Objectives:**

To assess potential risk factors for vertigo and dizziness in adolescents and to evaluate their variability by different vertigo types. The role of possible risk factors for vertigo and dizziness in adolescents and their population relevance needs to be addressed in order to design preventive strategies.

**Study design:**

The study population consisted of 1482 school-children between the age of 12 and 19 years, who were instructed to fill out a questionnaire on different vertigo types and related potential risk factors. The questionnaire specifically asked for any vertigo, spinning vertigo, swaying vertigo, orthostatic dizziness, and unspecified dizziness. Further a wide range of potential risk factors were addressed including gender, stress, muscular pain in the neck and shoulder region, sleep duration, migraine, coffee and alcohol consumption, physical activity and smoking.

**Results:**

Gender, stress, muscular pain in the neck and shoulder region, sleep duration and migraine were identified as independent risk factors following mutual adjustment: The relative risk was 1.17 [1.10–1.25] for female sex, 1.07 [1.02–1.13] for stress, 1.24 [1.17–1.32] for muscular pain, and 1.09 [1.03–1.14] for migraine. The population attributable risk explained by these risk factors was 26%, with muscular pain, stress, and migraine accounting for 11%, 4%, and 3% respectively.

**Conclusion:**

Several established risk factors in adults were also identified in adolescents. Risk factors amenable to prevention accounted for 17% of the total population risk. Therefore, interventions targeting these risk factors may be warranted.

## Introduction

In general population dizziness and vertigo symptoms constitute a very common complaint with a lifetime prevalence for dizziness of up to 30% and for vertigo of up to 10% [1]. Several risk factors favouring the occurrence of vertigo or dizziness have been consistently observed in adult or elderly populations. Vertigo is often associated to migraine [2], female gender [3, 4] and psychological disorders such as depression [5, 6] and anxiety [7]. Although vertigo and dizziness complaints in adolescents are as common as in adults [8], to our knowledge there is only limited data about potential risk factors in adolescents and children. A couple of studies with main focus on general health perception in adolescents have reported that females experienced dizziness more often in the last 6 months than males [9, 10]. One further study of 31 children between 6 and 17 years of age described a significant association of vertigo with headache [11]. One limitation of these studies though is, that they do not distinguish between different vertigo and/or dizziness types.

The present study in 1482 grammar school students (age range: 12 to 19 years) aimed to delineate potential risk factors for the occurrence of different types of vertigo and dizziness and to estimate their population attributable risk fractions in adolescents.

## Methods

### Population

The study population for this cross-sectional study was drawn from the headache interventions study MUKIS (**M**ünchner **U**ntersuchung zu **K**opfschmerzen bei Gymnasiasten–**I**nterventions-**S**tudie), which is described in detail elsewhere [12]. For this study, 12 out of 47 public grammar schools in the greater area of Munich, Germany, agreed to participate. Reasons for non-participation were its related administrative work load and inability to organize the study in the school setting.

The study was conducted between November 2011 and January 2012 when a member of the author team instructed the students to fill out a questionnaire during a visit of the specific school. In total 1661 students attending the 8^th^, 9^th^, and 10^th^ school grade filled out the questionnaire and a total of 1482 students (mean age: 14.47±1.08, range 12–19 years) completed the full questionnaire and were included for the analysis.

The study was approved by the Data Safety Officer and the Ethics Committee of the Medical Faculty of the Ludwig-Maximilians-University Munich and the Bavarian Ministry for Teaching and Culture. All participating students and their parents/guardians gave written informed consent to participate in the study.

### Assessment of vertigo and vertigo types

Period prevalence of dizziness/vertigo was assessed by the question “Did you suffer from dizziness or vertigo during the last 3 months?” which could be answered with “yes” or “no”. If answered “yes”, students were asked to further specify the vertigo type: “spinning vertigo like in a carrousel” (spinning vertigo), “swaying vertigo like on a small boat” (swaying vertigo), “feeling of impending black out when rapidly standing up” (orthostatic dizziness), or “neither of the three types” (unspecified dizziness).

### Assessment of body positions and movements related to vertigo types

The body positions and movements related to different vertigo types were assessed by the following questions, which could be answered with “yes” or “no”: The vertigo is a) triggered or aggravated by head movements, b) triggered by change of position (e.g. standing up from laying), c) also present when sitting or lying down, d) only present when standing or walking.

### Assessment of potential risk factors

Chronic stress was assessed by the global stress index, which is based on 12 of the 57 items included in the TICS (Trier Inventory of Chronic Stress) [13]. The frequency within the past 3 months for each of these items was indicated on a 5-point rating scale from 0 (never) to 4 (very often). Individual mean scores of all 12 items were calculated and t-transformed based on age-group specific norm values (mean = 50; SD = 10). For the individual t-value, it was determined whether the value was within normal range of the t-distribution, below or above average. Chronic stress was assumed when the value was above the normal range. A good reliability (Cronbach´s alpha of the global stress index: 0.91), high construct validity and high scale correlation has been found [13].

Muscular pain in the neck and shoulder region was assessed by the question “Do you suffer from muscle pain in the head, neck and shoulder region?” which could be answered with “yes” or “no”.

Coffee drinkers were defined as those reported drinking at least one cup of coffee per week. Alcohol consumption was defined by consuming at least one glass of beer, wine/champagne, or cocktails/liquor per month. The definition of coffee drinkers and of alcohol consumption was selected according to Milde-Busch et al. [14], who found associations of high coffee and alcohol consumption with headache in adolescents. Physical activity was assessed by questions on the frequency of weekly physical activity outside of school hours. Metabolic equivalent indices of the reported activities were calculated and categorized as high, moderate, or low by sample-specific tertiles from the distribution according to the procedure suggested by Kujala et al. [15]. Students in the lowest tertile were assumed to be physically inactive, and the others as physically active. Students were labeled as “smoking” when answering to the question “Do you smoke?” with “yes”. Students were asked about their usual sleep duration (in hours). The average sleep duration per day was calculated and dichotomized into “>8 h sleep per day” and “≤ 8 h sleep per day”. This dichotomy was chosen according to the recommendations for sleep duration of the American Academy of Sleep Medicine [16].

For the assessment of migraine students were asked about suffering from any headache in the last 6 months. When answering with “yes”, questions on headache symptoms were used to classify the headache type based on criteria set by the International Classification of Headache Disorders– 3rd edition (ICHD-III beta) [17] as either migraine, tension type headache, probable migraine, probable tension type headache and miscellaneous headache as described in a previous publication [18].

### Statistical methods

Body positions and movements (head movements, change of position, sitting or lying, standing or walking) related to vertigo types (orthostatic dizziness, spinning vertigo, swaying vertigo and unspecified dizziness) were analyzed by calculating the proportion of students reporting a specific body position/movement and the corresponding 95%-confidence intervals (95%-CI) in the four groups of vertigo types.

Prevalence with 95%-CI of any vertigo and vertigo types by exposure to risk factors (gender, chronic stress, muscular pain, coffee consumption, alcohol consumption, physical inactivity, smoking, short sleep duration, and migraine) were calculated. The association among the identified risk factors was assessed in phi correlation tests. In univariate log-binomial regression model unadjusted risk ratios with 95%-CIs were calculated for the association between the identified risk factors and vertigo. To account for the intercorrelation between risk factors, independent associations for associated risk factors for vertigo were assessed in mutual adjusted log-binomial regression models. Risk ratios with 95% CIs were estimated.

To assess the potential impact of the identified risk factors on a population level, population attributable risk fractions (PARF) were estimated. This estimate considers not only the strength of the association, but additionally the prevalence of exposure in the investigated population. Model-based approaches like the average attributable fraction [19] are considered the most robust estimators for the PARF. We used codes for the statistical software R provided by Rückinger et al. [20] to calculate the PARF with this method.

## Results

The mean age of the students who took part in the present study was 14.48 years (SD = 1.08) ranging from 12 to 19 years of age. 55% (N = 809) were female and 45% (N = 673) were male. A detailed analysis of the prevalence of vertigo and dizziness in the MUKIS study has been published previously [8]. In summary, a total 72% of the student reported some kind of vertigo/dizziness in the last of 3 months. From these 52% suffered from orthostatic dizziness, 12% from spinning vertigo, 12% from swaying vertigo and 16% of unspecified dizziness, with some of the students reporting more than one kind of vertigo symptom.

### Body positions and movements related to vertigo types

Change of position was the most prevalent factor for triggering vertigo without a consistent difference between the vertigo types. A relation to head movements and sitting or lying was mainly observed in spinning and swaying vertigo. All vertigo types were equally reported to be present when standing or walking by about half of the students (Table 1).

**Table 1:** Body positions and movements as a trigger for different vertigo types.

	Vertigo triggered or aggravated by head movements	Vertigo triggered by change of position	Vertigo also present when sitting or lying	Vertigo only present when standing or walking
	%			
	[95%CI]			
**Orthostatic dizziness**	41.9	87.2	22.7	40.7
	[38.0–45.8]	[84.5–89.5]	[19.5–26.3]	[36.8–44.7]
**Spinning vertigo**	56.6	73.4	47.6	47.4
	[48.3–64.6]	[64.9–80.6]	[38.9–56.6]	[39.0–56.0]
**Swaying vertigo**	58.6	79.7	46.8	46.0
	[50.4–66.3]	[72.3–85.6]	[38.5–55.3]	[37.9–54.3]
**Unspecified dizziness**	37.8	75.8	24.5	41.0
	[30.9–45.2]	[69.3–81.3]	[18.5–31.7]	[33.9–48.5]

CI = confidence interval

### Risk factors for vertigo and different vertigo types

Coffee and alcohol consumption, smoking, and physical activity were not associated with vertigo (see Table in S1 File). Table 2 delineates results on the risk factors significantly associated with the occurrence of vertigo in general and the different vertigo types. Female students reported vertigo more often than male students with respect to all vertigo types except for spinning vertigo and unspecific vertigo. Further, all vertigo types were significantly associated with increased stress as indicated by an elevated TICS score except for unspecified vertigo. Also, patients with muscular pain in the neck and shoulder region reported significantly more often to suffer from all vertigo types except for unspecified vertigo. A reduced sleep duration (<8hours/day) was only associated with swaying vertigo. Finally, patients diagnosed with migraine experienced significantly more often vertigo, especially orthostatic dizziness and swaying vertigo.

**Table 2:** Prevalence of vertigo and specific vertigo types in students exposed/unexposed to potential risk factors.

Risk factor		Vertigo	Vertigo Types			
		N = 1066				
			Orthostatic dizziness	Spinning vertigo	Swaying vertigo	Unspecified dizziness
			N = 766	N = 173	N = 179	N = 232
		%	%	%	%	%
		[95%-CI]	[95%-CI]	[95%-CI]	[95%-CI]	[95%-CI]
		(N)	(N)	(N)	(N)	(N)
**Gender**	**Female**	**79.3**	**57.9**	12.3	**15.2**	17.6
	**N = 809**					
		**[76.3–82.0]**	**[54.4–61.3]**	[10.2–14.8]	**[12.8–17.9]**	[15.1–20.5]
		**(642)**	**(469)**	(100)	**(123)**	(143)
	**Male**	**63**	**44.1**	10.8	**8.3**	13.2
		**[59.2–66.6]**	**[40.3–47.9]**	[8.6–13.5]	**[6.4–10.7]**	[10.8–16.0]
	**N = 673**					
		**(424)**	**(297)**	(73)	**(56)**	(89)
**Chronic Stress–global stress index from TICS**^**1**^	**Above normal range**	**85.7**	**63.2**	**17.9**	**21.3**	19.0
	**N = 351**	**[81.5–89.1]**	**[57.9–68.2]**	**[14.1–22.4]**	**[17.2–26.1]**	[15.1–23.6]
		**(301)**	**(222)**	**(63)**	**(75)**	(67)
	**Within normal range**	**67.6**	**48.1**	**9.7**	**9.2**	14.5
	**N = 1131**	**[64.8–70.3]**	**[45.1–51.0]**	**[8.0–11.6]**	**[7.6–11.0]**	[12.6–16.8]
		**(765)**	**(544)**	**(110)**	**(104)**	(165)
**Muscular pain in the neck and shoulder region**	**Yes**	**83.3**	**59.9**	**14.3**	**17.8**	17.8
	**N = 656**					
		**[80.2–86.1]**	**[56.0–63.6]**	**[11.7–17.3]**	**[15.0–21.0]**	[15.0–21.0]
		**(547)**	**(393)**	**(94)**	**(117)**	(117)
	**No**	**62.8**	**45.1**	**9.5**	**7.5**	13.9
	**N = 826**					
		**[59.4–66.1]**	**[41.7–48.6]**	**[7.6–11.8]**	**[5.8–9.5]**	[11.6–16.5]
		**(519)**	**(373)**	**(79)**	**(62)**	(115)
**Short sleep duration**	**in average <8 hours**	75.4	54.7	13.4	**16.1**	14.4
	**N = 497**	[71.3–79.1]	[50.2–59.1]	[10.6–16.8]	**[13.0–19.6]**	[11.5–17.9]
		(375)	(272)	(67)	**(80)**	(72)
	**in average ≥8 hours**	70.1	50.1	10.7	**10.0**	16.2
	**N = 985**	[67.1–72.9]	[46.9–53.3]	[8.9–12.9]	**[8.2–12.1]**	[14.0–18.7]
		(691)	(494)	(106)	**(99)**	(160)
**Migraine**	**Yes**	**84.7**	**62.2**	15.2	**19.2**	16.4
	**N = 249**					
		**[79.5–88.8]**	**[55.8–68.2]**	[11.1–20.4]	**[14.6–24.8]**	[12.2–21.7]
		**(211)**	**(155)**	(38)	**(48)**	(41)
	**No**	**69.3**	**49.5**	10.9	**10.6**	15.4
	**N = 1233**					
		**[66.6–71.8]**	**[46.7–52.3]**	[9.2–12.8]	**[8.9–12.5]**	[13.5–17.6]
		**(855)**	**(611)**	(135)	**(131)**	(191)

N = number; CI = confidence interval. Significant differences (non-overlapping 95%-CIs) are printed in bold.

^1^TICS = Trier Inventory of Chronic Stress

### Univariate and mutually adjusted associations between identified risk factors and vertigo

Table 3 shows the univariate and mutually adjusted associations between the identified risk factors (gender, chronic stress, muscular pain, migraine) and vertigo. Unadjusted risk ratios ranged from 1.22 for migraine to 1.35 for muscular pain. As all identified risk factors were significantly associated among each other (p<0.001 for all combinations), a mutually adjusted model was calculated. Following adjustment, independent associations were observed for all risk factors. Muscular pain appeared to be the strongest independent risk factor for vertigo.

**Table 3:** Relative risk for vertigo by gender, chronic stress, muscular pain and migraine.

	Any vertigo	
	Univariate model	Mutually adjusted model
	RR	RR
	[95%-CI]	[95%-CI]
**Gender (female)**	**1.26**	**1.17**
	**[1.18–1.35]**	**[1.10–1.25]**
**Chronic stress–global stress index from TICS**^**1**^ **(Above normal range)**	**1.27**	**1.07**
	**[1.20–1.34]**	**[1.02–1.13]**
**Muscular pain in the neck and shoulder region**	**1.33**	**1.24**
	**[1.25–1.41]**	**[1.17–1.32]**
**Migraine**	**1.22**	**1.09**
	**[1.15–1.30]**	**[1.03–1.14]**

### Population attributable risk fractions (PARF)

Table 4 shows the PARF of the identified risk factors. In total these risk factors accounted for 26% of the population risk. Muscular pain, chronic stress, and migraine are possibly amenable to preventive interventions. The proportions of the burden of suffering from vertigo related to these risk factors amounted to 17%.

**Table 4:** Population attributable risk fraction of independent risk factors.

Risk factors	PARF (%)
Gender (female)	8.75
Chronic stress–global stress index from TICS^1^ (Above normal range)	4.22
Muscular pain in the neck and shoulder region	10.64
Migraine	2.76
Total	26.37

## Discussion

In an adolescent population of considerable size and a high burden of suffering from vertigo, we assessed a broad range of possible risk factors. Several risk factors reported in adults could be confirmed in adolescents including female sex, stress, muscular pain in the neck and shoulder region, and migraine, as well as a reduced sleep duration (only for swaying vertigo). After mutual assessment, because of intercorrelation of these risk factors, the risk increment related to these factors was in the range of 7 to 24%. The potential impact of removal of the risk factors amenable to prevention (chronic stress, muscular pain and migraine) amounted to 17%.

### Gender specificity

The present study shows a significant association of vertigo with female sex in an age group between 12–19 years, which matches data for older populations [4, 21–23]. There is only limited data on gender differences in adolescents concerning vertigo diseases. Two of three studies in adolescents have reported a female predominance in general health complaints including the feeling of being dizzy [9, 10], whereas one retrospective study failed to identify gender differences in adolescents <18 years [24]. The strength of the effect in these studies, reported as odds ratios, ranged between 0.39 and 1.79. Our observation matches the female gender-specificity reported for most common vertigo diseases in adolescents, such as vestibular migraine [11, 24–26].

The significant association of female sex with vertigo was mainly explained by the vertigo types orthostatic dizziness and swaying vertigo. Orthostatic dizziness has been shown to be a common symptom in adult populations with the prevalence ranging between 5 to 13% [27]. Interestingly both studies showed the highest prevalence of orthostatic dizziness in the youngest age group <29 years reaching up to 22%. Further both studies show a female predominance of orthostatic dizziness. Orthostatic dizziness can result from a diversity of pathologies such as initial orthostatic hypotension (IOH), chronic orthostatic intolerance, dysautonomic orthostatic intolerance (orthostatic hypotension), and postural orthostatic tachycardia syndrome (POTS) including exercise intolerance [28, 29]. The most common etiology in children and adolescents is considered to be IOH [30–32], although no detailed epidemiological data is available. IOH is a benign form of orthostatic intolerance with autonomic integrity that rarely leads to unconsciousness [30, 32]. One risk factor for IOH is considered to be a asthenic habitus [31]. It may be accepted that female students more often correspond to this kind of habitus and thus explain the female predominance in the present study. Further POTS has been shown to have a female predominance in adolescents [33, 34]. A possible explanation for lower orthostatic tolerance in women is suggested by Fu et al. [35]. While sympathetic neural responses during orthostasis are similar in men and women, Fu et al. found a smaller stroke volume due to lower cardiac filling in women. They postulate that this may overwhelm the vasomotor reserve for vasoconstriction or precipitate neutrally mediated sympathetic withdrawal. Unfortunately, the questionnaire used in the present study could not differentiate between possible underlying pathologies.

Swaying vertigo is often described as a symptom of somatoform and/or phobic vertigo [36] and in lesser extend of vestibular migraine [37]. In general somatoform disorders among adolescents are considered to be common [38, 39]. Reports on gender differences in somatoform disorders of adolescents though remain controversial [40]. While some studies report no gender differences in the prevalence of somatoform disorders in adolescents [39, 41] others do [38, 42]. Data on somatoform vertigo in adolescents is sparse, although it seems to be one of the most frequent diagnosis among adolescents with vertigo symptoms [43]. Only one study reports a female predominance in somatoform vertigo [44], which matches the findings of the present study. A general increased sensitivity of reporting body sensations [42, 45] and/or a different reporting style of somatoform symptoms [46] have been suggested to result into such an female predominance.

In migraine, a female predominance starting in puberty is generally accepted [47–50]. This is mainly explained by hormonal changes as numerous studies have shown associations between headache and the menstrual cycle [51–53]. Similarly, such a female predominance is also seen in vestibular migraine in adults [2], as well as in adolescents [44], which matches the findings of the present study. As specific studies on the etiology of such gender differences in vestibular migraine are missing, only similar mechanisms as in classical migraine can be postulated.

### Muscular pain

In adults muscular pain and vertigo symptoms often coexist [54–56]. To our knowledge this is the first study to show such an association in an adolescent population. Muscular pain was the strongest identified risk factor for vertigo (see Table 3), accounting for 11% of the vertigo risk in the study population (see Table 4). In clinical practice the coexistence of neck and shoulder pain with vertigo has often the consequence that the vertigo is attributed to the cervical spine and diagnosed as “cervical vertigo” [57–59], which in the literature constitutes a highly debatable diagnosis [58, 60, 61]. Proprioceptive afferences, mostly originating from the neck`s muscle spindles, have been suggested to project to vestibular nuclei and modify their activity according to position changes of the head [62]. In consequence, a disturbed proprioception from the cervical segments could lead to a vertigo sensation [61]. Interestingly the distribution, morphology, and density of muscle spindles in muscles of the cervical spine seem not to change with age [63], so that the mechanisms that may lead to “cervical vertigo” could be similar in adolescents and adults. “Cervical vertigo” as an independent entity though remains a matter of debate and in many cases the vertigo symptoms can be attributed to a number of differential diagnoses [58, 60, 61].

Further associations of muscular pain in the neck and shoulder region with vertigo and/or dizziness have been shown in whiplash associated disorders [64, 65] and in degenerative cervical disorders [66]. As degenerative disorders do not play a role in adolescents, whiplash injuries could occur in adolescents. Unfortunately, the questionnaire used in the present study did not include questions about trauma history.

An alternative explanation of the coexistence of vertigo and muscular pain in the neck and shoulder region could be that the cervical muscular pain occurs reactively to the vertigo, e.g. mediated by stress. Psychological factors including stress can lead to acute and chronic pain conditions in adults [67] as well as in adolescents [68, 69]. Additionally, patients with somatoform disorders, which are common among adolescents with vertigo complaints [43, 44], also frequently experience muscular pain [70], so that an underlying somatoform disorder could further explain the association of vertigo and muscular pain. Finally it is notable, that migraine is also known to be associated to muscular pain in the neck and shoulder region [71, 72]. Interestingly the association for muscular pain and vertigo even persisted after adjustment for stress and migraine, suggesting that muscular pain constitutes an independent risk factor. The exact pathophysiology of this association remains unknown and poses an interesting field for future research. Nevertheless, interventions leading to a reduction of muscular pain might also reduce the burden of vertigo. Similarly, these patients might also benefit from stress prevention and effective migraine treatment.

### Stress & sleep duration

Stress is known to be associated with vertigo and dizziness in older study groups [73], as well as in younger groups [74]. Similarly to the present study, Torsheim and Wold [74] found a strong association between school-related stress and dizziness symptoms in an age group of 11–15 years. The strength of the effect in this study, with odds ratios up to 5.4, is considerably higher than in our study. However, the authors used the odds ratio to describe the relative risk, which overestimates the strength of the effect for common outcomes and used an unspecific outcome (“dizziness”). The causal direction between stress and vertigo is inconclusive. Several authors suggest that stress leads to vertigo symptoms[73], while others argue for a reversed causation [75]. While dizziness and vertigo symptoms in children are as common as in adults [8], underlying vestibular disorders in children seem to be rare [43, 76]. Therefore, the causal direction that stress leads to vertigo symptoms seems to be more likely. This is also supported by the elevated TICS score in students experiencing swaying vertigo, a typical characteristic of somatoform vertigo or vestibular migraine [36, 37], which has also been shown to be strongly associated to stress [77–79].

Further swaying vertigo was the only vertigo type that showed a significant association with sleep duration. It is known that reduced sleep duration in adolescents can lead, among others, to complaints such as dizziness [80, 81]. Sleep deprivation in adolescents can also be associated with stress in general [81–83], suggesting a possible bi-directional interaction between sleep duration and stress with vertigo. However, the relative risk for sleep deprivation changed only marginally after adjustment for stress (data not shown), suggesting that stress is not in the causal pathway.

### Migraine

In 35% [11] to 60% [84] of adolescents vertigo symptoms have been reported to accompany headaches. Especially adolescents with migraine, the second most common headache type in adolescents [85], 15% to 25% also experience some kind of vertigo or dizziness [44, 86, 87]. Similarly, in the present study 85% of all students fulfilling the criteria of migraine headache also experienced some kind of vertigo. Unfortunately, the questionnaire structure did not allow distinguishing the temporal sequence of the occurrence of headache and vertigo properly. Therefore, we cannot say whether both symptoms were a coincidence and which preceded which. When looking at the vertigo types individually, orthostatic dizziness showed the strongest association with migraine. Studies show that migraine patients more often suffer from orthostatic problems and syncopes than the healthy population [88]. It has been postulated that dopamine hypersensitivity contributes to the pathophysiology of migraine leading to a dopamine mediated inhibition of norepinephrine release, which then again results into hypotension [89]. This could explain the significant correlation of migraine with orthostatic dizziness in the present study.

### Contribution of the risk factors to the total population risk of vertigo

The contribution of the individual risk factors to the population risk is explained by the strength of the effect and by the prevalence of the risk factor: a weak risk factor may be highly relevant on the population level if common, while a strong risk factor may be almost irrelevant if the risk factor is rare. The contribution of the risk factors can be expressed by the PARF. In total, the identified risk factors accounted for 26% of the risk of vertigo in adolescents. The proportion of risk factors potentially amenable to preventive interventions amounted to 17%. The most promising risk factors to be targeted, with respect to total population risk, were muscular pain and stress. This may allow establishing preventive therapies in school children by early mediation of coping strategies and if possible stress prevention. For example, the effectiveness of a one-time, classroom-based prevention program for headache could be demonstrated by a previous publication within the scope of the MUKIS study [12].

### Strength and limitations of the study

The strength of the study is the population size and the analysis of specific vertigo types. Unlike most previous studies, we took account of interdependencies between the risk factors by mutual adjustment. Further by assessing the PARF we identified the proportion of the potential total risk reduction of vertigo by preventive strategies.

The limitations of the present study are similar to most cross-section questionnaire studies. E.g., reversed causation for muscular pain or stress and vertigo cannot be ruled out. A further limitation, with respect to conceptualize interventions, may be, that we did not further analyze the factors causing stress in school children. But it is known from previous studies that factors such as school work, social contacts, and bullying constitute main factors for self-perceived stress [90].

## Conclusion

In conclusion, female gender, muscular pain in the neck and shoulder region, stress, sleep duration, and migraine seem to be significant risk factors for the development of different dizziness or vertigo types in adolescents between 12 and 19 years of age. Following mutual adjustment because of intercorrelation among these risk factors, the strength of these associations was modest. These risk factors are nevertheless relevant, because they are common in adolescents. On the population level, the total PARF for risk factors amenable to prevention amounted to 17%, so that preventive strategies appear to be warranted.

## Supporting information

S1 File Table. Prevalence of vertigo and specific vertigo types in students exposed/unexposed to potential risk factors, that were not significantly associated with vertigo/vertigo types. (DOCX)

S2 File Dataset. (CSV)

S3 File Questionnaire in German (original language). (DOC)

S4 File Questionnaire in English. (DOC)
