# Supplementary material for: Vertigo and dizziness in adolescents: Risk factors and their population attributable risk
Source: PLoS One. 2017 Nov 13;12(11):e0187819. doi: 10.1371/journal.pone.0187819 (PMC5683632; doi:10.1371/journal.pone.0187819)
Supplement: S1 File — (DOCX) [file pone.0187819.s001.docx]

**S1 Table.** Prevalence of vertigo and specific vertigo types in students exposed/unexposed to potential risk factors, that were not significantly associated with vertigo/vertigo types.

| **Risk factor** | | **No vertigo** | **Vertigo** | **Vertigo Types** | | | |
| --- | --- | --- | --- | --- | --- | --- | --- |
|  |  |  | **N=1066** |  |  |  |  |
|  |  | **N=416** |  |  |  |  |  |
|  |  |  |  | **Orthostatic dizziness** | **Spinning vertigo** | **Swaying vertigo** | **Unspecified dizziness** |
|  |  |  |  | **N=766** | **N=173** | **N=** **179** | **N=232** |
|  |  | % | % | % | % | % | % |
|  |  | [95%-CI] | [95%-CI] | [95%-CI] | [95%-CI] | [95%-CI] | [95%-CI] |
|  |  | (N) | (N) | (N) | (N) | (N) | (N) |
| **Coffee consump-tion** | **≥1 cup per week** | 26.5 | 73.4 | 54.2 | 11.3 | 13.4 | 16.4 |
|  |  | [ 23.2 - 30.1 ] | [ 69.8 - 76.7 ] | [ 50.2 - 58.0 ] | [ 9.0 - 14.0 ] | [ 10.9 - 16.3 ] | [ 13.7 - 19.6 ] |
|  | **N=655** | ( 174 ) | ( 481 ) | ( 355 ) | ( 74 ) | ( 88 ) | ( 108 ) |
|  | **<1 cup per week** | 29.2 | 70.7 | 49.7 | 11.9 | 11.0 | 14.9 |
|  |  | [ 26.2 - 32.5 ] | [ 67.4 - 73.8 ] | [ 46.2 - 53.1 ] | [ 9.8 - 14.4 ] | [ 8.9 - 13.3 ] | [ 12.6 - 17.6 ] |
|  | **N=827** | ( 242 ) | ( 585 ) | ( 411 ) | ( 99 ) | ( 91 ) | ( 124 ) |
| **Alcohol consump-tion** | **≥1-3 glasses per week**  **N=757** | 26.4 | 73.5 | 54.0 | 12.9 | 12.5 | 15.4 |
|  |  | [ 23.3 - 29.7 ] | [ 70.2 - 76.6 ] | [ 50.4 - 57.6 ] | [ 10.6 - 15.6 ] | [ 10.3 - 15.1 ] | [ 12.9 - 18.2 ] |
|  |  | ( 200 ) | ( 557 ) | ( 409 ) | ( 98 ) | ( 95 ) | ( 117 ) |
|  | **< 1 glas per week** | 29.7 | 70.2 | 49.2 | 10.3 | 11.5 | 15.8 |
|  |  | [ 26.5 - 33.2 ] | [ 66.7 - 73.4 ] | [ 45.5 - 52.9 ] | [ 8.2 - 12.8 ] | [ 9.3 - 14.2 ] | [ 13.3 - 18.7 ] |
|  | **N=725** | ( 216 ) | ( 509 ) | ( 357 ) | ( 75 ) | ( 84 ) | ( 115 ) |
| **Physical inactivity** | **Yes** | 25.4 | 74.5 | 54.1 | 11.8 | 12.7 | 13.2 |
|  |  | [ 21.0 - 30.2 ] | [ 69.7 - 78.9 ] | [ 48.8 - 59.3 ] | [ 8.8 - 15.7 ] | [ 9.5 - 16.6 ] | [ 10.0 - 17.2 ] |
|  | **N=362** |  |  |  |  |  |  |
|  |  | ( 92 ) | ( 270 ) | ( 196 ) | ( 43 ) | ( 46 ) | ( 48 ) |
|  | **No** | 28.9 | 71.0 | 50.8 | 11.6 | 11.8 | 16.4 |
|  |  | [ 26.3 - 31.7 ] ( 324 ) | [ 68.3 - 73.6 ] ( 796 ) | [ 47.9 - 53.8 ] ( 570 ) | [ 9.8 - 13.6 ]  ( 130 ) | [ 10.0 - 13.9 ] ( 133 ) | [ 14.3 - 18.7 ] ( 184 ) |
|  | **N=1120** |  |  |  |  |  |  |
| **Smoking** | **Yes** | 25.9 | 74.0 | 53.3 | 14.4 | 12.9 | 16.8 |
|  |  | [ 20.2 - 32.5 ] | [ 67.4 - 79.7 ] | [ 46.3 - 60.2 ] | [ 10.0 - 20.1 ] | [ 8.8 - 18.5 ] | [ 12.1 - 22.7 ] |
|  | **N=208** |  |  |  |  |  |  |
|  |  | ( 54 ) | ( 154 ) | ( 111 ) | ( 30 ) | ( 27 ) | ( 35 ) |
|  | **No** | 28.4 | 71.5 | 51.4 | 11.2 | 11.9 | 15.4 |
|  |  | [ 25.9 - 30.9 ] | [ 69.0 - 74.0 ] | [ 48.6 - 54.1 ] | [ 9.5 - 13.1 ] | [ 10.2 - 13.8 ] | [ 13.5 - 17.5 ] |
|  | **N=1274** |  |  |  |  |  |  |
|  |  | ( 362 ) | ( 912 ) | ( 655 ) | ( 143 ) | ( 152 ) | ( 197 ) |
