# Supplementary material for: Vertigo and dizziness in adolescents: Risk factors and their population attributable risk
Source: PLoS One. 2017 Nov 13;12(11):e0187819. doi: 10.1371/journal.pone.0187819 (PMC5683632; doi:10.1371/journal.pone.0187819)
Supplement: S3 File — (DOC) [file pone.0187819.s003.doc]

| Institut für Soziale Pädiatrie und Jugendmedizin | Kinderklinik und Poliklinik im  Dr. von Haunerschen Kinderspital | Klinikum Großhadern  Neurologische Klinik  und Poliklinik |
| --- | --- | --- |
| Abteilung für Epidemiologie im Kindes- und Jugendalter  Prof. Dr. Rüdiger von Kries | Abteilung für Pädiatrische Neurologie | Entwicklungsneurologie | Sozialpädiatrie | iSPZ^LMU^  Prof. Dr. Florian Heinen | Oberbayerisches Kopfschmerzzentrum  Prof. Dr. Andreas Straube |
| Heiglhofstraße 63 • 81377 München  Telefon 089-71009-314 • Telefax 089-71009 315  ruediger.kries@med.uni-muenchen.de | Lindwurmstraße 4 • 80337 München  Telefon 089-5160-7851 • Telefax 089-5160 7745  florian.heinen@med.uni-muenchen.de | Marchioninistraße 15 • 81377 München  Telefon 089-7095-3900 • Telefax 089-7095-3677  andreas.straube@med.uni-muenchen.de |

**MUKIS: MÜNCHNER UNTERSUCHUNG ZU KOPFSCHMERZEN BEI GYMNASIASTEN - INTERVENTIONSSTUDIE**

Klasse-Nummer: [klasse] ______ Schule-Nummer: [schule] _______ Datum: [datum/start] _____

**Vorbemerkung Erstbefragung**

Kopfschmerzen sind häufig – besonders bei jugendlichen Gymnasiasten. Sie lassen sich möglicherweise vermeiden – ob und wie ist das Ziel dieser Untersuchung. Hierzu werden zunächst die Häufigkeit von Kopfschmerzen und mögliche Risikofaktoren erhoben. Bitte füllen Sie dafür den Fragebogen aus. In etwa 6 bis 8 Monaten werden wir Sie noch einmal befragen, um die Stabilität der Symptome zu überprüfen. In der Zwischenzeit wird eine Beratung zu Kopfschmerzen angeboten, die in einigen Klassen bereits heute, in anderen Klassen erst in 6 bis 8 Monaten erfolgt.

Alle Informationen, die wir in dieser Studie erheben, werden streng vertraulich behandelt und nicht für den Einzelfall, sondern nur in Gruppen zusammengefasst ausgewertet.

Bitte beantworten Sie die Fragen so genau und offen wie möglich. Es gibt keine falschen oder richtigen Antworten. Machen Sie bitte an keiner Stelle Angaben zu anderen Personen (z. B. bei Sonstige oder Andere), dies würde zum Ausschluss des Fragebogens führen. Für unsere Auswertungen ist es sehr wichtig, dass Sie alle Fragen vollständig beantworten. Wir bedanken uns für Ihre Mitarbeit bei diesem wichtigen Forschungsprojekt. Viel Spaß bei der Beantwortung!

**Ihr persönlicher Code**

Damit wir Ihre Daten in pseudonymisierter Form analysieren können, bekommt jeder seinen eigenen Code, den nur Sie kennen. Bitte beantworten Sie hierfür die folgenden fünf Fragen und tragen jeweils den entsprechenden Buchstaben ein. [code]

- Der dritte Buchstabe Ihres Vornamens lautet: _ _ _
- Der letzte Buchstabe Ihres Nachnamens lautet: _ _ _
- Der erste Buchstabe Ihres Geburtsortes lautet: _ _ _
- Der dritte Buchstabe des Vornamens Ihrer Mutter lautet: _ _ _
- Der letzte Buchstabe Ihres Vornamens lautet: _ _ _

**Fragen zur Person**

**1. Sind Sie …** [sex] männlich? ………………  [1] weiblich? ………………  [2]

**2. Wie alt sind Sie?** [alter] __ __ Jahre

**3. Wie würden Sie Ihren Gesundheitszustand im Allgemeinen beschreiben?**  [gesallg]

Sehr gut ….…………………………………………………………………  [1]

Gut ……………………….…………………………………………………  [2]

Mittelmäßig ..………………………………………………………………  [3]

Schlecht ..…………………..………………………………………………  [4]

Sehr schlecht ...……………………………………………………………  [5]

**4. Sind Sie mit Ihrer gesundheitlichen Situation zufrieden?**  [geszufr]

Ja ….………………………………………………………………….………  [1]

Nein ……………………….…………………………………………………  [0]

Weiß nicht …………...………….…………………….……………....….…  [2]

**Fragen zu Schmerzen und Beschwerden**

**5. Hatten Sie Kopfschmerzen?**  [ksallg]

Nein .………………………..………………………………………………  [0] weiter bei Frage 16

Ja, in den vergangenen 7 Tagen ..………………………………………  [1]

Ja, in den vergangenen 3 Monaten ..……………………………………  [2]

Ja, in den vergangenen 6 Monaten ..……………………………………  [3]

Ja, in den vergangenen 12 Monaten ……………………………………  [4]

**6. Wie lange dauern Ihre Kopfschmerzen an, wenn Sie keine Schmerzmittel einnehmen oder eine Behandlung erfolglos bleibt?**  [ksdauer]

Bis 30 Minuten .……………..………………………………………………  [1]

30 Minuten bis 1 Stunde ...……………..…………………………………  [2]

1 bis 4 Stunden ……………………………………………………………  [3]

4 Stunden bis 3 Tage ..……………………………………………………  [4]

3 bis 7 Tage ..………………………………………………………………  [5]

Mehr als 7 Tage ..…………………………………………………………  [6]

Weiß nicht .…………………………………………………………………  [7]

**7. Wie oft sind diese Kopfschmerzen bei Ihnen mindestens schon aufgetreten?**

[ksfreq]

Weniger als 5 Mal …………………………………………………………  [1]

5 bis 9 Mal .…………………………………………………………………  [2]

10 Mal oder häufiger ...……………………………………………………  [3]

**8. Wie lange leiden Sie schon an solchen Kopfschmerzen? Geben Sie bitte die entsprechende Zeit in Monaten oder Jahren an.**

__ __ Monate [ksmonat] __ __ Jahre [ksjahr]

**9. Haben Sie Kopfschmerzen, …**

**Ja Nein**

… die sich auf eine Kopfhälfte beschränken? [kstyp01] ..………………………………  [1]  [0]

… die bei Ihnen beidseitig auftreten? [kstyp02] …………………………………………  [1]  [0]

… die einen pulsierenden oder pochenden Charakter haben? [kstyp03] .………………  [1]  [0] … die einen dumpfen, drückenden bis ziehenden Charakter haben? [kstyp04] ……  [1]  [0]

… die Ihre üblichen Tagesaktivitäten erheblich beeinträchtigen? [kstyp05] ……………  [1]  [0]

… die durch körperliche Aktivität (z.B. …) verstärkt werden? [kstyp06] ..…  [1]  [0]

… die von Übelkeit begleitet werden? [kstyp07] ……………………………………………  [1]  [0]

… die von Erbrechen begleitet werden? [kstyp08] …………………………………………  [1]  [0]

… die von Schwindel begleitet werden? [kstyp09] …………………………………………  [1]  [0]

… die von Geräuschüberempfindlichkeit begleitet werden? [kstyp10] …………………  [1]  [0]

… die von Lichtüberempfindlichkeit begleitet werden? [kstyp11] ………………………  [1]  [0]

… die von Geruchsempfindlichkeit begleitet werden? [kstyp12] ………………..………  [1]  [0]

… bei denen es vorher zu … (Flimmern) kommt? [kstyp13] ……………………………..  [1]  [0]

… bei denen es vorher zu … Gefühlsstörungen kommt? [kstyp14] ……………….……  [1]  [0]

… bei denen es vorher zu für Minuten anhaltenden Schwindel kommt? [kstyp15] ……  [1]  [0]

… nur für Mädchen: die zusammen mit der Regelblutung auftreten? [kstyp16] ……  [1]  [0]

**10. Wenn Sie an die Tage denken, an denen Sie in den letzten 3 Monaten Kopfschmerzen hatten, wie würden Sie die durchschnittliche Stärke der Schmerzen einstufen?**  [ksstark]

**Ganz schwach Mittel Ganz stark**

1 2 3 4 5 6 7 8 9 10

[1] [2] [3] [4] [5] [6] [7] [8] [9] [10]

**11. An wie vielen Tagen in den letzten 3 Monaten …**

… hatten Sie Kopfschmerzen? .………………………………………………….... __ __ Tage [kstage01]

… konnten Sie wegen der Kopfschmerzen nicht zur Schule gehen? ............... __ __ Tage [kstage02]

… konnten Sie darüber hinaus wegen der Kopfschmerzen nur teilweise

am Schulunterricht teilnehmen? ………………………………………..… __ __ Tage [kstage03]

… konnten Sie darüber hinaus wegen der Kopfschmerzen nur halb so viel

leisten? ..……………………………………………………………..……… __ __ Tage [kstage04]

… konnten Sie wegen der Kopfschmerzen zu Hause nichts tun? ………....... __ __ Tage [kstage05]

… mussten Sie wegen der Kopfschmerzen auf sportliche oder andere

Freizeitaktivitäten verzichten? …………………………………………… __ __ Tage [kstage06]

… haben Sie zwar an Freizeitaktivitäten teilgenommen, aber konnten nur

halb so viel leisten? ………………………………………………..……… __ __ Tage [kstage07]

**12. Wie oft haben Sie in den letzten 3 Monaten wegen Ihrer Kopfschmerzen Schmerzmittel eingenommen?**  [ksmittelfreq]

Nie ...………………………..………………………………………………  [0]

Selten ..……………………..………………………………………………  [1]

Gelegentlich ...……………..………………………………………………  [2]

Fast jedes Mal ……………..………………………………………………  [3]

Jedes Mal …………………..………………………………………………  [4]

**13. Wie behandeln Sie Ihre Kopfschmerzen sonst? (Mehrfachantworten möglich)**

Gar nicht, abwarten [ksbehand01] ………..………………………...……………  [1]

Schlafen/sich hinlegen, Ruhe, Dunkelheit [ksbehand02] ….………………......  [1]

Etwas trinken [ksbehand03]…………………………………………..…..…….…  [1]

Etwas essen [ksbehand04] ………………………………….………...…………...  [1]

Medikamente [ksbehand05] …………………………….………...………....……  [1]

Nasses/kaltes Tuch auf den Kopf legen [ksbehand06] …....……………….......  [1]

Bewegung, Spazierengehen, frische Luft [ksbehand07] ..……………..........  [1]

Entspannungsverfahren [ksbehand08] .…………………………..…….......…  [1]

Kopfmassage, Akupressur, Schläfen massieren [ksbehand09] .......................  [1]

Musik, Ablenkung [ksbehand10] …………………………...……...……..………  [1]

Pfefferminzöl auf Stirn/Schläfen auftragen [ksbehand11] …………….........…  [1]

Trinken von Kaffee, Cola oder Energydrinks [ksbehand12] …………...….......  [1]

Anders [ksbehand13] ……….…..………………………………..……………......  [1]

**14. Waren Sie in den letzten 12 Monaten wegen Ihrer Kopfschmerzen beim Arzt?**

[ksarzt]

Nein .………………………..………………………………………………  [0] weiter bei Frage 16

Ja .………………………………… ………………………………….……  [1] weiter bei Frage 15

**15. Hat der Arzt bei Ihnen eine der folgenden Diagnosen festgestellt?**  [ksdiagn]

Spannungskopfschmerz…..………………………………………………  [1]

Migräne …….……………..……………………………….………………  [2]

Doppeldiagnose: Spannungskopfschmerz + Migräne ……..…………  [3]

Keine dieser Diagnosen…..………………………………………………  [0]

**16. Was denken Sie, wodurch werden (Ihre) Kopfschmerzen ausgelöst? (Mehrfachantworten möglich)**

Stress, Überforderung, Belastung [ksausloes01] ……………............…………  [1]

Viel Schularbeiten, schulischer Druck [ksausloes02] ……....................………  [1]

Streit, sozialer Stress [ksausloes03] .…………………….……….....……...……  [1]

Durch Krankheit, Verletzung … Erkältung, Grippe [ksausloes04] ............…..  [1]

Muskuläre Verspannungen im … -Schulter-Bereich [ksausloes05] .…………  [1]

Regelblutung [ksausloes06] .……..………..……………………...…………….…  [1]

Falsche Sehstärke/Brille [ksausloes07] .………………………....……….………  [1]

Zu wenig Schlaf/Müdigkeit [ksausloes08] ...….………..….....………..…………  [1]

Zu wenig getrunken/Flüssigkeitsmangel [ksausloes09] …....…………..………  [1]

Ungesunde Ernährung, zu viel/zu wenig gegessen [ksausloes10] …….………  [1]

Zu viel Alkohol, „Kater“ [ksausloes11] …………………………...………….…… [1]

Rauchen, Drogen [ksausloes12] ..……………………………………...……….…  [1]

Zu viel Sport [ksausloes13] ..….……………………………………………….…… [1]

Zu wenig Bewegung [ksausloes14] ………..………………………………...……. [1]

Zu lange Beschäftigung mit TV, PC, iPad [ksausloes15] ....…………………  [1]

Lärm, laute Musik [ksausloes16] ……………………….……………...…………  [1]

Wetter/Klima (z. B. …), Luftdruck, Jahreszeiten [ksausloes17] …...................  [1]

Keine frische Luft [ksausloes18] …………………………………………..…..…  [1]

Zu viel Kaffee, Cola oder Energydrinks [ksausloes19] ………..……............…  [1]

Vererbt [ksausloes20] …………….……………………….………………………  [1]

Weiß nicht [ksausloes21] .………………………………………...………...…..…  [1]

Sonstige [ksausloes22]…………………………………………….……….........…  [1]

**17. Haben Sie, ohne dass Sie etwas Besonderes tun, schmerzhafte Muskeln im Kopf-/Hals-/ Schulterbereich?**  [mukhs]

Nein .………………………..………………………………………………  [0] weiter bei Frage 19

Ja ……………………………………………………………………………  [1] weiter bei Frage 18

**18. Markieren Sie diese Stellen schmerzhafter Muskeln bitte in der Skizze mit einem „X“.**

**19. Tasten oder spüren Sie besonders feste/verhärtete, druck- oder schmerzempfindliche Stellen in den Muskeln des Kopf-/Hals-/Schulterbereichs?**  [drukhs]

Nein .………………………..………………………………………………  [0] weiter bei Frage 21

Ja ……………………………………………………………………………  [1] weiter bei Frage 20

**20. Markieren Sie diese Stellen bitte in der Skizze mit einem „X“.**

**21. Ich leide unter folgenden Beschwerden**

**Stark Mäßig Kaum Gar nicht**

Schwächegefühl [bl01] ...……………………………………  [3] …...  [2] ……  [1] ...…  [0]

Stiche, Schmerzen oder Ziehen in der Brust [bl02] ..……  [3] …...  [2] ……  [1] ...…  [0]

Druck- oder Völlegefühl im Leib [bl03] .…………………  [3] …...  [2] ……  [1] ...…  [0]

Mattigkeit [bl04] ……………………………………………  [3] …...  [2] ……  [1] ...…  [0]

Übelkeit [bl05] ..……………………………………………  [3] …...  [2] ……  [1] ...…  [0]

Reizbarkeit [bl06] .…………………………………………  [3] …...  [2] ……  [1] ...…  [0]

Grübelei [bl07] .………………………………………………  [3] …...  [2] ……  [1] ...…  [0]

Starkes Schwitzen [bl08] ……………………………………  [3] …...  [2] ……  [1] ...…  [0]

Kreuz- oder Rückenschmerzen [bl09] .……………………  [3] …...  [2] ……  [1] ...…  [0]

Innere Unruhe [bl10] ..………………………………………  [3] …...  [2] ……  [1] ...…  [0]

Schweregefühl bzw. Müdigkeit in den Beinen [bl11] ..…  [3] …...  [2] ……  [1] ...…  [0]

Unruhe in den Beinen [bl12] .………………………………  [3] …...  [2] ……  [1] ...…  [0]

Überempfindlichkeit gegen Kälte [bl13] .…………………  [3] …...  [2] ……  [1] ...…  [0]

Übermäßiges Schlafbedürfnis [bl14] ……………………  [3] …...  [2] ……  [1] ...…  [0]

Schlaflosigkeit [bl15] ..………………………………………  [3] …...  [2] ……  [1] ...…  [0]

Schwindelgefühl [bl16] ……………………………………  [3] …...  [2] ……  [1] ...…  [0]

Zittern [bl17] .………………………………………………  [3] …...  [2] ……  [1] ...…  [0]

Nacken- oder Schulterschmerzen [bl18] .…………………  [3] …...  [2] ……  [1] ...…  [0]

Bauchschmerzen [bl19] .……………………………………  [3] …...  [2] ……  [1] ...…  [0]

Nur für Mädchen: Regelbeschwerden [bl20] .……………  [3] …...  [2] ……  [1] ...…  [0]

**22. Hatten Sie in den letzten 3 Monaten Schwindel?**  [schwallg]

Nein .………………………..……………………………………………..….……  [0] w. b. Frage 28

Ja, Schwindel/“Schwarzwerden vor den Augen“ bei zu raschem Aufstehen…  [1]

Ja, Drehschwindel wie im Karussell……………………...………….……………  [2]

Ja, Schwankschwindel wie auf einem kleinen Boot …..……………....………  [3]

Ja, aber weder Drehschwindel, Schwankschwindel noch

„Schwarzwerden vor den Augen“ bei zu raschem Aufstehen ..…………….…  [4]

**23. War der Schwindel…**

**Ja Nein**

… auslösbar/verstärkt durch Kopfbewegungen? [schwwann01] ..………………………  [1]  [0]

… auslösbar durch Lageänderung (z. B. Aufstehen aus dem Liegen)? [schwwann02]  [1]  [0]

… auch im Liegen und Sitzen (in Ruhe) vorhanden? [schwwann03].……………….……  [1]  [0]

… nur beim Stehen und Gehen (bei Bewegungen) vorhanden? [schwwann04]...………  [1]  [0]

**24. Wenn Sie Schwindel hatten, wurde dieser von folgenden Beschwerden begleitet? …Immer Manchmal Nie**

Kopfschmerzen/Kopfdruck/Nackenschmerzen? [schwbegleit01] …….….…  [2]…  [1]…  [0]

Hörstörungen/Ohrdruck/Ohrgeräusche? [schwbegleit02] ……….…….....…  [2]…  [1]…  [0]

Kribbeln an Armen, Beinen oder im Gesicht? [schwbegleit03] ………..……  [2]…  [1]…  [0]

Sehstörungen (… Flimmern)? [schwbegleit04] ……………………....…....…  [2]…  [1]…  [0]

**25. Wie lange dauert der Schwindel bei Ihnen an?**  [schwdauer]

Bis 1 Minute …………………..….…………………………………………  [1]

1 bis 30 Minuten ……………….………..…………………………………  [2]

30 Minuten bis 4 Stunden …………………………………………………  [3]

4 bis 24 Stunden ……………………………………………………………  [4]

1 bis 7 Tage …………………………………………………………………  [5]

Mehr als 7 Tage …….………………………………………………………  [6]

Weiß nicht .……………………….…………………………………………  [0]

**26. Wie oft ist dieser Schwindel bei Ihnen in den vergangenen 12 Monaten aufgetreten?**

[schwfreq]

1 Mal ……………………………………………………...…………………  [1]

Weniger als 5 Mal .…………………………...……………………………  [2]

Mehr als 5 Mal ...……………………………………………………………  [3]

**27. Wie stark waren Sie wegen des Schwindels eingeschränkt? Konnten Sie …**

**Ja Nein**

… wegen des Schwindels nicht zur Schule gehen? [schwstaerke01] ……………………  [1]  [0]

… wegen des Schwindels nicht Ihren Freizeitaktivitäten nachgehen? [schwstaerke02]  [1]  [0]

… wegen des Schwindels nur liegen und nicht aufstehen? [schwstaerke03] …………  [1]  [0]

**Fragen zu Lebensumständen**

**28. Bitte beantworten sie die folgenden Fragen danach, wie häufig Sie die darin angesprochene Erfahrung gemacht bzw. Situation erlebt haben. Denken Sie bei der Beantwortung bitte an die vergangenen 3 Monate und versuchen Sie sich daran zu erinnern, wie oft Sie in diesem Zeitraum die jeweilige Erfahrung gemacht haben. Bei einigen Schilderungen geht es um Arbeit, denken Sie dabei bitte an Schule und Schularbeiten.**

**Bitte beantworten Sie alle Fragen der Reihe nach, ohne eine auszulassen.**

|  | **Nie Selten Manchmal Oft Immer** |
| --- | --- |
| Befürchtung, dass … Unangenehmes passiert [tics01] … | [0]  [1] …  [2]  [3]  [4] |
| Zeiten, in denen ich sorgenvolle Gedanken **nicht** unterdrücken kann [tics02] ……………………………… | [0]  [1] …  [2]  [3]  [4] |
| Ich bemühe mich vergeblich, mit guten Leistungen Anerkennung zu erhalten [tics03] ...…………………… | [0]  [1] …  [2]  [3]  [4] |
| Zeiten, in denen mir die Sorgen über … wachsen [tics04] | [0]  [1] …  [2]  [3]  [4] |
| Obwohl ich mein Bestes gebe, wird meine Arbeit nicht gewürdigt [tics05] ..……………………………………… | [0]  [1] …  [2]  [3]  [4] |
| Zeiten, in denen ich nicht die Leistung bringe, die von mir erwartet wird [tics06] ….………………………………… | [0]  [1] …  [2]  [3]  [4] |
| Zeiten, in denen ich mir viele Sorgen mache und nicht damit aufhören kann [tics07] .…...………….…………… | [0]  [1] …  [2]  [3]  [4] |
| Zeiten, in … zu viele Verpflichtungen zu erfüllen habe [tics08] | [0]  [1] …  [2]  [3]  [4] |
| Zeiten, in … mir die Arbeit über den Kopf wächst [tics09] | [0]  [1] …  [2]  [3]  [4] |
| Befürchtung, meine Aufgaben nicht erfüllen zu … [tics10] | [0]  [1] …  [2]  [3]  [4] |
| Erfahrung, dass alles zu viel ist, was ich zu tun habe [tics11] | [0]  [1] …  [2]  [3]  [4] |
| Zeiten, in denen mir die Verantwortung für andere zur Last wird [tics12] …...…………………………………… | [0]  [1] …  [2]  [3]  [4] |

**Fragen zu Ernährungs- und Lebensgewohnheiten**

**29. Wie viel koffeinhaltigen Kaffee trinken Sie zurzeit? (1 Tasse = 200 ml)**  [kafkof]

Gar keinen koffeinhaltigen Kaffee .......………………  [0]

1 Tasse pro Woche …..…………………………………  [1]

Täglich 1 Tasse …………………………………………  [2]

2 und mehr Tassen pro Tag .…………………………  [3]

**30. Wie viel koffeinhaltige Getränke (z. B. „Energydrinks“, Cola) trinken Sie zurzeit? (1 Glas = 200 ml)**

[getrkof]

Gar keine .....…………….…………………….…………………....….……...  [0]

1 Glas pro Woche ………………………………………………....…..…..…  [1]

Täglich 1 Glas …………………...…………………………………..……...…  [2]

Mehr als 2 Gläser (bzw. eine 0,5 l-Flasche und mehr) pro Tag .........…  [3]

**31. Wie viel alkoholische Getränke trinken Sie zurzeit?**

**Bier und Wein, Obstwein Andere Mixgetränke,**

**Biermixgetränke und Sekt Cocktails und Schnaps**

[alkbier] [alkwein] [alkmix]

Gar keine ………………….….…….  [0] ……..………  [0] ………………  [0]

1 bis 3 Gläser pro Monat …..………  [1] ……..………  [1] ………………  [1]

1 Glas pro Woche ...….……………  [2] ……..………  [2] ………………  [2]

Mehrere Gläser pro Woche ….……  [3] ……..………  [3] ………………  [3]

**32. Wie oft treiben Sie in Ihrer Freizeit (außerhalb des Schulunterrichts) Sport?**  [sportoft]

Gar nicht ...………………………………………………………….………  [0]

Weniger als 1 Mal pro Monat ..………………..…………………………  [1]

1 bis 3 Mal pro Monat ..……………………………………………………  [2]

1 Mal pro Woche ..…………………………………………………………  [3]

2 Mal pro Woche ..…………………………………………………………  [4]

3 Mal pro Woche ..…………………………………………………………  [5]

4 Mal pro Woche ..…………………………………………………………  [6]

5 Mal pro Woche ..…………………………………………………………  [7]

6 Mal pro Woche ..…………………………………………………………  [8]

7 Mal und häufiger pro Woche ..……………………….…………………  [9]

**33. Sind Sie Mitglied in einem Sportverein oder Fitness-Studio?**  [verein]

Nein .………………………………………………………….………………  [0]

Ja, in einem ………………..…….…………………………..………………  [1]

Ja, in zwei bzw. beidem …...……….………………………………………  [2]

Ja, in drei oder mehr ………………………………………………………  [3]

**34. Wie lange treiben Sie jedes Mal im Durchschnitt während Ihrer Freizeit Sport?**

[sportdauer]

Kürzer als 11 Minuten ...………………………………………..…………  [0]

11 bis 20 Minuten ...……………………………………………..…………  [1]

21 bis 30 Minuten ...………………………………………….……………  [2]

31 bis 40 Minuten ...………………………………………….……………  [3]

41 bis 50 Minuten ...………………………………………….……………  [4]

51 bis 70 Minuten ...………………………………………….……………  [5]

71 bis 90 Minuten ...………………………………………….……………  [6]

Länger als 90 Minuten ...………………………………………….………  [7]

**35. Sind Ihre sportlichen Aktivitäten im Durchschnitt so anstrengend wie …**  [sportanstr]

… Spazierengehen? .………………………………………………………  [1]

… Jogging/Ausdauerlauf (lange Strecke, gemäßigtes Tempo)? …...…  [2]

… Laufen/Rennen? .………………………………………………………  [3]

… schnelles Laufen/Rennen/Kurzstreckenlauf (hohes Tempo)? .……  [4]

**36. Rauchen Sie zurzeit?**  [rauch]

Nein .………………………..………………………………………………  [0]

Ja, seltener als ein Mal pro Woche ………………………………………  [1]

Ja, ein Mal pro Woche .……………………………………………………  [2]

Ja, mehrmals pro Woche ...………………………………………………  [3]

Ja, täglich ..…………………………………………………………………  [4]

**37. Wie lange schlafen Sie normalerweise an Schultagen?** __ __ Stunden [schlafwo]

**38. Wie lange schlafen Sie normalerweise an Wochenend- oder Feiertagen?** __ __ Stunden [schlafwe]

**39. Welche Medikamente nehmen Sie regelmäßig ein?**

Keine [medik01] ………………..…………………………………………….……  [1]

Mittel gegen Asthma (z. B. Asthmaspray) [medik02] …………………..……  [1]

Mittel gegen Allergien (z. B. … Nasenspray) [medik03] …………………….  [1]

Mittel gegen ADHS [medik04] ……………………………………………………  [1]

Schlafmittel [medik05] ……………………………………………………….……  [1]

Schmerzmittel [medik06] …………………………………………………………  [1]

Andere [medik07] ………………………..…………………………………….……  [1]

**Fragen zum Hintergrund**

**40. Welchen höchsten Bildungsabschluss haben Ihre Eltern?**

**Vater Mutter**

[bildva] [bildmu]

Fach-/Hochschul-/Universitätsabschluss …….……………………..……  [1] ..…...……  [1]

Fachhochschulreife/Abitur (Gymnasium oder erweiterte Oberschule)  [2] ..…...……  [2]

Realschulabschluss/Mittlere Reife/POS 10. Klasse .......………………  [3] ..…...……  [3]

Haupt-/Volksschulabschluss ………………………………………………  [4] ..…...……  [4]

Schule beendet ohne Schulabschluss ……………………………………  [5] ..…...……  [5]

Weiß nicht .…………………………………………………………………  [0] ..…...……  [0]

**41. Welche Aussagen zur Berufstätigkeit treffen auf Ihre Eltern zu?**

**Vater Mutter**

[berufva] [berufmu]

Zurzeit nicht berufstätig (z. B. Hausfrau, Hausmann, Rentner) ..……  [1] ..…...……  [1]

Arbeitslos ..…………………………………………………………………  [2] ..…...……  [2]

Vorübergehende Freistellung (z. B. Erziehungsurlaub) ………………  [3] ..…...……  [3]

Teilzeit oder stundenweise berufstätig ...………………………………  [4] ..…...……  [4]

Voll berufstätig ……………………………………………………………  [5] ..…...……  [5]

Umschulung/Studium .……………………………………………………  [6] ..…...……  [6]

**Vielen Dank für Ihre Teilnahme und Mithilfe!**
