# Supplementary material for: Vertigo and dizziness in adolescents: Risk factors and their population attributable risk
Source: PLoS One. 2017 Nov 13;12(11):e0187819. doi: 10.1371/journal.pone.0187819 (PMC5683632; doi:10.1371/journal.pone.0187819)
Supplement: S4 File — (DOC) [file pone.0187819.s004.doc]

| Institut für Soziale Pädiatrie und Jugendmedizin | Kinderklinik und Poliklinik im  Dr. von Haunerschen Kinderspital | Klinikum Großhadern  Neurologische Klinik  und Poliklinik |
| --- | --- | --- |
| Abteilung für Epidemiologie im Kindes- und Jugendalter  Prof. Dr. Rüdiger von Kries | Abteilung für Pädiatrische Neurologie | Entwicklungsneurologie | Sozialpädiatrie | iSPZ^LMU^  Prof. Dr. Florian Heinen | Oberbayerisches Kopfschmerzzentrum  Prof. Dr. Andreas Straube |
| Heiglhofstraße 63 • 81377 München  Telefon 089-71009-314 • Telefax 089-71009 315  ruediger.kries@med.uni-muenchen.de | Lindwurmstraße 4 • 80337 München  Telefon 089-5160-7851 • Telefax 089-5160 7745  florian.heinen@med.uni-muenchen.de | Marchioninistraße 15 • 81377 München  Telefon 089-7095-3900 • Telefax 089-7095-3677  andreas.straube@med.uni-muenchen.de |

**MUKIS: MUNICH INVESTIGATION OF HEADACHE IN SECONDARY SCHOOL STUDENTS – INTERVENTIONAL STUDY**

Class number: [klasse] ______ School number: [schule] _______ Date: [datum/start] _____

**Introduction to the first questionnaire**

Headache is a frequent complaint – particularly in teenage secondary school students. It may be possible to avoid headache and this study aims to investigate whether and how this can be done. The first step is to record the headache frequency and possible risk factors. To do this, please fill out the questionnaire. In around 6 to 8 months we will survey you again to check the stability of the symptoms. In the meantime, you will be offered counselling about headache; in some classes this will take place today, in others in 6 to 8 months.

All the information we collect in this study will be strictly confidential and will be analysed by group, not by individual cases.

Please answer the questions as precisely and honestly as possible. There are no right or wrong answers. Please do not provide any information about other people (e.g. in “Other”). This would lead to the questionnaire being excluded from the study. For our analysis, it is very important that you answer all questions completely. Thank you for your help in this important research project. Have fun answering the questionnaire!

**Your personal code**

So that we can analyse your data in a pseudonymized way, you each receive your own code that only you know. To create this code, please answer the following five questions by entering one letter for each answer. [code]

- The third letter of your first name is: _ _ _
- The last letter of your last name is: _ _ _
- The first letter of your place of birth is: _ _ _
- The third letter of your mother’s first name is: _ _ _
- The last letter of your first name is: _ _ _

**Questions about you**

**1. Are you …** [sex] male? ………………  [1] female? ………………  [2]

**2. How old are you?** [alter] __ __ years

**3. How would describe your general state of health?**  [gesallg]

Very good ….………………………………………………………………  [1]

Good ……………………….………………………………………………  [2]

Fair ..………………………………………………………………  [3]

Bad ..…………………..………………………………………………  [4]

Very bad ...……………………………………………………………  [5]

**4. Are you happy about your state of health?**  [geszufr]

Yes ….……………………………………………………………….………  [1]

No ……………………….…………………………………………………  [0]

Don’t know …………...………….…………………………………....….…  [2]

**Questions about pain and ailments**

**5. Have you ever had a headache?**  [ksallg]

No .………………………..………………………………………………  [0] proceed to question 16

Yes, in the last week ..………………………………………  [1]

Yes, in the last 3 months ..……………………………………  [2]

Yes, in the last 6 months ..……………………………………  [3]

Yes, in the last 12 months ……………………………………  [4]

**6. How long does your headache last if you don’t take any painkillers or if another treatment is unsuccessful?** [ksdauer]

Up to 30 minutes .……………..……………………………………………  [1]

30 minutes to 1 hour ...……………..…………………………………  [2]

1 to 4 hours ……………………………………………………………  [3]

4 hours to 3 days ..……………………………………………………  [4]

3 to 7 days ..………………………………………………………………  [5]

More than 7 days ..…………………………………………………………  [6]

Don’t know .…………………………………………………………………  [7]

**7. How often have you had this headache?**

[ksfreq]

Fewer than 5 times …………………………………………………………  [1]

5 to 9 times .…………………………………………………………………  [2]

10 or more times ...……………………………………………………  [3]

**8. How long have you been suffering from these headaches? Please give the length of time in months or years.**

__ __ months [ksmonat] __ __ years [ksjahr]

**9. Do you have a headache …**

**Yes No**

… which occurs on only one side of your head? [kstyp01] ..………………………………  [1]  [0]

… which occurs on both sides of your head? [kstyp02] ……………………………………  [1]  [0]

… which is pulsating or throbbing? [kstyp03] .………………  [1]  [0] … which is dull and pressing? [kstyp04] ………………………………………..…  [1]  [0]

… which considerably affects your normal daily activities? [kstyp05] ……………  [1]  [0]

… which increases with physical activity (e.g. …)? [kstyp06] ..…  [1]  [0]

… which is accompanied by nausea? [kstyp07] ……………………………………………  [1]  [0]

… which is accompanied by vomiting? [kstyp08] …………………………………………  [1]  [0]

… which is accompanied by dizziness? [kstyp09] …………………………………………  [1]  [0]

… which is accompanied by hypersensitivity to noise? [kstyp10] …………………  [1]  [0]

… which is accompanied by hypersensitivity to light? [kstyp11] ………………………  [1]  [0]

… which is accompanied by hypersensitivity to smells? [kstyp12] ……………..………  [1]  [0]

… which is preceded by … (flickering)? [kstyp13] ……………………………..  [1]  [0]

… which is preceded by … disturbances of sensation? [kstyp14] ……………….……  [1]  [0]

… which is preceded by dizziness lasting minutes? [kstyp15] ……  [1]  [0]

… only for girls: which occurs with your period? [kstyp16] ……  [1]  [0]

**10. Thinking about days in the last 3 months on which you had a headache, how would you rate the average intensity of the pain?**  [ksstark]

**Very weak Moderate Very strong**

1 2 3 4 5 6 7 8 9 10

[1] [2] [3] [4] [5] [6] [7] [8] [9] [10]

**11. On how many days in the last 3 months …**

… did you have a headache? .………………………………………………….... __ __ days [kstage01]

… were you not able to go to school because of headache? ........................... __ __ days [kstage02]

… were you – in addition to the days mentioned in the previous question – only partly able to take part in lessons because of headache? ………………………………………..… __ __ days [kstage03]

… were you only able to perform half as well because of headache? .……… __ __ days [kstage04]

… were you not able to do anything at home because of headache? ……....... __ __ days [kstage05]

… did you have to forego sport or other leisure activities because of headache? … __ __ days [kstage06]

… did you take part in leisure activities, but were only able to perform half as well? ………………………………………………..……… __ __ days [kstage07]

**12. How often in the last 3 months have you taken painkillers because of headache?**  [ksmittelfreq]

Never ...………………………..……………………………………………  [0]

Rarely ..……………………..………………………………………………  [1]

Occasionally ...……………..………………………………………………  [2]

Nearly every time …………..………………………………………………  [3]

Every time …………………..………………………………………………  [4]

**13. How else do you treat your headache? (Tick all applicable answers)**

I don’t, I just wait for it to go away [ksbehand01] ………………...……………  [1]

Sleeping/lying down, rest, darkness [ksbehand02] ……..………………......  [1]

Drinking [ksbehand03]…………………………………………..…..…….…  [1]

Eating [ksbehand04] ………………………………….………...…………...  [1]

Drugs [ksbehand05] ………..…………………….………...………....……  [1]

Placing a wet/cold cloth on your head [ksbehand06] …....……………….......  [1]

Movement, going for a walk, fresh air [ksbehand07] ..……………..........  [1]

Relaxation techniques [ksbehand08] .…………………………..…….......…  [1]

Head massage, acupressure, massaging temples [ksbehand09] ....................  [1]

Music, distraction [ksbehand10] …………………………...……...……..………  [1]

Applying peppermint oil to forehead/temples [ksbehand11] ……….........…  [1]

Drinking coffee, cola or energy drinks [ksbehand12] …………...….......  [1]

Other [ksbehand13] ……….…..…..……………………………..……………......  [1]

**14. Have you been to the doctor in the last 12 months because of headache?**

[ksarzt]

No .………………………..………………………………………………  [0] proceed to question 16

Yes .………………………………… ………………………………….……  [1] proceed to question 15

**15. Did the doctor diagnose you with one the following?**  [ksdiagn]

Tension headache…..………………………………………………  [1]

Migraine …….……………..……………………………….………………  [2]

Double diagnosis: tension headache + migraine ……..…………  [3]

None of these diagnoses…..………………………………………………  [0]

**16. What do you think triggers your headache? (Tick all applicable answers)**

Stress, mental overload, pressure [ksausloes01] ……………............…………  [1]

Lots of homework, pressure at school [ksausloes02] ……....................………  [1]

Arguments, social stress [ksausloes03] .…………………….….....……...……  [1]

Illness, injuries … colds, flu [ksausloes04] ......................................…..  [1]

Muscular tension in … shoulder area [ksausloes05] .……………..……  [1]

Menstruation [ksausloes06] .……..………..……………………...…………….…  [1]

Incorrect prescription for glasses/contacts [ksausloes07] .…....……….………  [1]

Not enough sleep/tiredness [ksausloes08] ...….……..….....………..…………  [1]

Not drunk enough fluids/dehydration [ksausloes09] …....…………..…………  [1]

Unhealthy diet, eating too much/too little [ksausloes10] …….…………  [1]

Too much alcohol, hangover [ksausloes11] ….…………………...………….…… [1]

Smoking, drugs [ksausloes12] ..…………………………………..…...……….…  [1]

Too much exercise [ksausloes13] .…………………………………………….…… [1]

Too little exercise [ksausloes14] ………..……………………….…………...……. [1]

Spending too long watching TV or on PC or iPad [ksausloes15] .……………  [1]

Noise, loud music [ksausloes16] ……………………….……………...…………  [1]

Weather/climate (e.g. …), air pressure, seasons [ksausloes17] …..................  [1]

No fresh air [ksausloes18] …………………………………………..…..…  [1]

Too much coffee, cola or energy drinks [ksausloes19] ………..……............…  [1]

Hereditary [ksausloes20] …………….…………………….………………………  [1]

Don’t know [ksausloes21] .……………………………………...………...…..…  [1]

Other [ksausloes22]…………………………..…………………….……….........…  [1]

**17. Do you have painful muscles in the head/neck/shoulder region with no obvious cause?**

[mukhs]

No .………………………..………………………………………………  [0] proceed to question 19

Yes …………………………………………………………………………  [1] proceed to question 18

**18. Please mark the areas with painful muscles with an “X”.**

**19. Can you feel particularly tight/hard, pressure-sensitive or tender areas in the muscles of the head/neck/shoulder region?**

[drukhs]

No .………………………..………………………………………………  [0] proceed to question 21

Yes …………………………………………………………………………  [1] proceed to question 20

**20. Please mark these areas with an “X”.**

**21. I suffer from the following**

**Severely Moderately Hardly Not at all**

Feeling of weakness [bl01] ...………………………………  [3] …...  [2] ……  [1] ...…  [0]

Twinges or pain in the chest [bl02] ..……  [3] …...  [2] ……  [1] ...…  [0]

Abdominal pressure or fullness [bl03] .…………………  [3] …...  [2] ……  [1] ...…  [0]

Exhaustion [bl04] ……………………………………………  [3] …...  [2] ……  [1] ...…  [0]

Nausea [bl05] ..……………………………………………  [3] …...  [2] ……  [1] ...…  [0]

Irritability [bl06] .…………………………………………  [3] …...  [2] ……  [1] ...…  [0]

Brooding [bl07] .……………………………………………  [3] …...  [2] ……  [1] ...…  [0]

Excess perspiration [bl08] …………………………………  [3] …...  [2] ……  [1] ...…  [0]

Back pain [bl09] .……………………  [3] …...  [2] ……  [1] ...…  [0]

Feeling of restlessness [bl10] ..……………………………  [3] …...  [2] ……  [1] ...…  [0]

Heavy or tired legs [bl11] ..…  [3] …...  [2] ……  [1] ...…  [0]

Restless legs [bl12] .………………………………  [3] …...  [2] ……  [1] ...…  [0]

Hypersensitivity to cold [bl13] .…………………  [3] …...  [2] ……  [1] ...…  [0]

Excessive need for sleep [bl14] ……………………  [3] …...  [2] ……  [1] ...…  [0]

Sleeplessness [bl15] ..………………………………………  [3] …...  [2] ……  [1] ...…  [0]

Feeling of dizziness [bl16] …………………………………  [3] …...  [2] ……  [1] ...…  [0]

Trembling [bl17] .……………………………………………  [3] …...  [2] ……  [1] ...…  [0]

Neck or shoulder pain [bl18] .…………………  [3] …...  [2] ……  [1] ...…  [0]

Stomach ache [bl19] .……………………………………  [3] …...  [2] ……  [1] ...…  [0]

Only for girls: menstrual disorders [bl20] .……………  [3] …...  [2] ……  [1] ...…  [0]

**22. Have you experienced dizziness in the last 3 months?**  [schwallg]

No .………………………..………………………………………..….……  [0] proceed to question 28

Yes, dizziness/feeling faint when standing up too quickly…  [1]

Yes, rotatory dizziness, like on a merry-go-round………….……………  [2]

Yes, postural dizziness, like on a small boat …..……………....………  [3]

Yes, but not rotatory dizziness, postural dizziness or

feeling faint when standing up too quickly ..…………….…  [4]

**23. Was the dizziness…**

**Yes No**

… triggered/intensified by head movements? [schwwann01] ..………………………  [1]  [0]

… triggered by changes in position (e.g. getting up from lying down)? [schwwann02]  [1]  [0]

… also present when lying or sitting (at rest)? [schwwann03].……………….……  [1]  [0]

… only present when standing or walking (when moving)? [schwwann04]...………  [1]  [0]

**24. If you experienced dizziness, was it accompanied by the following? Always Sometimes Never**

Headache/pressure in the head/neck pain? [schwbegleit01] …….….…  [2]…  [1]…  [0]

Hearing disturbances/pressure in the ears/tinnitus? [schwbegleit02] … [2]…  [1]…  [0]

Pins and needles in the arms, legs or face? [schwbegleit03] ………..…  [2]…  [1]…  [0]

Visual disturbances (… flickering)? [schwbegleit04] …………....…....…  [2]…  [1]…  [0]

**25. How long did the dizziness last?**  [schwdauer]

Up to 1 minute …………………..….………………………………………  [1]

1 to 30 minutes ……………….………..…………………………………  [2]

30 minutes to 4 hours…………………………………………………  [3]

4 to 24 hours ……………………………………………………………  [4]

1 to 7 days …………………………………………………………………  [5]

More than 7 days …….……………………………………………………  [6]

Don’t know .…………………….…………………………………………  [0]

**26. How often did the dizziness occur in the last 12 months?**

[schwfreq]

Once ……………………………………………………...………………  [1]

Fewer than 5 times …………………………...……………………………  [2]

More than 5 times ...………………………………………………………  [3]

**27. How greatly were you affected by the dizziness? Were you …**

**Yes No**

… not able to go to school because of the dizziness? [schwstaerke01] …………………  [1]  [0]

… not able to do your usual leisure activities because of the dizziness? [schwstaerke02]  [1]  [0]

… only able to lie down and not stand up because of the dizziness? [schwstaerke03] … [1]  [0]

**Questions about your personal circumstances**

**28. Please answer the following questions, indicating how often you experienced the situation mentioned. When answering, please think about the last 3 months and try to remember how often you experienced each situation in this period. Some of the statements refer to work; please think about school and homework here.**

**Please answer the questions in order without leaving any out.**

|  | **Never Rarely Sometimes Often Always** |
| --- | --- |
| Fear that … something bad will happen [tics01] … | [0]  [1] …  [2]  [3]  [4] |
| Times when I **can’t** suppress worried thoughts [tics02] ……………………………… | [0]  [1] …  [2]  [3]  [4] |
| I try in vain to win approval by performing well [tics03] ...…………………… | [0]  [1] …  [2]  [3]  [4] |
| Times when my worries become too much for me [tics04] | [0]  [1] …  [2]  [3]  [4] |
| Although I do my best, my work is not appreciated [tics05] ..……………………………………… | [0]  [1] …  [2]  [3]  [4] |
| Times when I don’t perform as well as is expected [tics06] ….………………………………… | [0]  [1] …  [2]  [3]  [4] |
| Times when I worry a lot and can’t stop worrying [tics07] .…...………….…………… | [0]  [1] …  [2]  [3]  [4] |
| Times when I have too many obligations [tics08] | [0]  [1] …  [2]  [3]  [4] |
| Times when work is too much for me [tics09] | [0]  [1] …  [2]  [3]  [4] |
| Fear of not fulfilling my duties and responsibilities … [tics10] | [0]  [1] …  [2]  [3]  [4] |
| Feeling that I have much too much to do [tics11] | [0]  [1] …  [2]  [3]  [4] |
| Times when responsibility for others becomes a burden for me [tics12] …...…………………………………… | [0]  [1] …  [2]  [3]  [4] |

**Questions on eating habits and lifestyle**

**29. How many caffeinated coffees do you drink at the moment? (1 cup = 200 ml)**  [kafkof]

No caffeinated coffee .......………………  [0]

1 cup per week …..…………………………………  [1]

1 cup per day …………………………………………  [2]

2 or more cups per day .…………………………  [3]

**30. How many caffeinated drinks (e.g. energy drinks, cola) do you drink at the moment? (1 glass = 200 ml)**

[getrkof]

None .....…………….……………………….…………………....….……...  [0]

1 glass per week ………………………………………………....…..…..…  [1]

1 glass per day …………………...…………………………………..……...…  [2]

More than 2 glasses (or more than one 500ml bottle) per day .........…  [3]

**31. How many alcoholic drinks do you drink at the moment?**

**Beer and Wine, fruit wine Other mixed drinks,**

**beer-based mixed and sparkling cocktails and spirits**

**drinks wine**

[alkbier] [alkwein] [alkmix]

None ………………….….…….  [0] ……..………  [0] ………………  [0]

1 to 3 glasses per month …..………  [1] ……..………  [1] ………………  [1]

1 glass per week ...….……………  [2] ……..………  [2] ………………  [2]

Several glasses per week ….……  [3] ……..………  [3] ………………  [3]

**32. How often do you exercise in your free time (outside of school sports lessons)?**  [sportoft]

Never ...………………………………………………………….………  [0]

Less than once a month ..………………..…………………………  [1]

1 to 3 times a month ..……………………………………………………  [2]

Once a week ..…………………………………………………………  [3]

Twice a week ..…………………………………………………………  [4]

3 times a week ..…………………………………………………………  [5]

4 times a week ..…………………………………………………………  [6]

5 times a week ..…………………………………………………………  [7]

6 times a week ..…………………………………………………………  [8]

7 or more times a week ..……………………….…………………  [9]

**33. Do you belong to a sports club or a gym?**  [verein]

No .………………………………………………………….………………  [0]

Yes, one of them ………………..…….…………………………..………………  [1]

Yes, two/both …...……….………………………………………  [2]

Yes, three or more ………………………………………………………  [3]

**34. How long on average do you exercise each time?**

[sportdauer]

Less than 11 minutes ...………………………………………..…………  [0]

11 to 20 minutes ...……………………………………………..…………  [1]

21 to 30 minutes ...………………………………………….……………  [2]

31 to 40 minutes ...………………………………………….……………  [3]

41 to 50 minutes ...………………………………………….……………  [4]

51 to 70 minutes ...………………………………………….……………  [5]

71 to 90 minutes ...………………………………………….……………  [6]

More than 90 minutes ...………………………………………….………  [7]

**35. Are your sporting activities on average as strenuous as …**  [sportanstr]

… going for a walk? .………………………………………………………  [1]

… jogging/endurance run (long distance, moderate pace)? …...…  [2]

… running? .………………………………………………………  [3]

… fast running/racing/sprint (high speed)? .……  [4]

**36. Do you smoke at the moment?**  [rauch]

No .………………………..………………………………………………  [0]

Yes, less than once a week ………………………………………  [1]

Yes, once a week .……………………………………………………  [2]

Yes, several times a week ...………………………………………………  [3]

Yes, daily ..…………………………………………………………………  [4]

**37. How long do you normally sleep on school nights?** __ __ hours [schlafwo]

**38. How long do you normally sleep at the weekend or on bank holidays?** __ __ hours [schlafwe]

**39. What medication do you take regularly?**

None [medik01] ………………..…………………………………………….……  [1]

Medication for asthma (e.g. asthma inhaler) [medik02] …………………..……  [1]

Medication for allergies (e.g. nasal spray) [medik03] …………………….  [1]

Medication for ADHS [medik04] …………………………………………………  [1]

Sleeping pills [medik05] …………………………………………………….……  [1]

Painkillers [medik06] …………………………………………………………  [1]

Other [medik07] ………………………..…………………………………….……  [1]

**Questions about your background**

**40. What is your parent’s highest educational qualification?**

**Father Mother**

[bildva] [bildmu]

Polytechnic/university degree …….……………………..……  [1] ..…...……  [1]

A levels ……………………..………………………….  [2] ..…...……  [2]

GCSEs .......…………………………………………  [3] ..…...……  [3]

Certificate of secondary education ………………………………………  [4] ..…...……  [4]

Left school with no qualifications ……………………………………  [5] ..…...……  [5]

Don’t know .…………………………………………………………………  [0] ..…...……  [0]

**41. Which of the following statements about jobs apply to your parents?**

**Father Mother**

[berufva] [berufmu]

Currently not employed (e.g. housewife, househusband, retired)  [1] ..…...……  [1]

Unemployed ..………………………………………………………………  [2] ..…...……  [2]

Temporary leave of absence (e.g. parental leave) ………………  [3] ..…...……  [3]

In part-time employment ...………………………………  [4] ..…...……  [4]

In full-time employment …………………………………………………  [5] ..…...……  [5]

Retraining/studying .……………………………………………………  [6] ..…...……  [6]

**Thank you for participating!**
